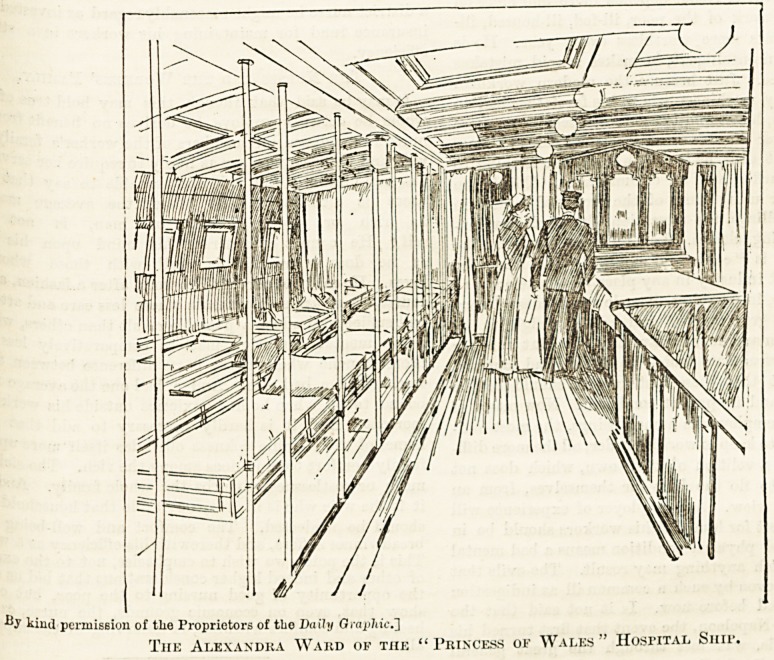# "The Hospital" Nursing Mirror

**Published:** 1900-03-03

**Authors:** 


### "The Hospital" Nursing Mirror.

Being the Nursing Section of "The Hospital."

[Contributions for this Section of "The Hospital" should be addressed to the Editor, The Hospital, 28 & 29, Southampton Street, Strand, London, W.C., and should have the word "Nursing" plainly written in left-hand top corner of the envelope.]

### Notes on News from the Nursing World.

### ROYAL VISITS TO NETLEY HOSPITAL.

Alike to patients and to nurses at Netley the events of the week have, of course, been the Royal visits to the Military Hospital. On Monday, after the Prince and Princess of Wales, who were accompanied by the Duke of York, had been to Southampton to see the sick and wounded soldiers on board the "Princess of Wales" hospital ship, they repaired to Netley, and addressed words of encouragement to the patients. On Tuesday the Queen herself spent nearly three hours in the wards, taking light and fragrance into the presence of between five and six hundred sufferers, to each of whom she individually addressed a word of gracious sympathy, and to most a bunch of flowers. As she was wheeled by her Indian attendant through the wards, on her mission of love, the thought must have occurred to many that even in her glorious reign she never had a more truly Royal progress.

### STILL MORE WAR NURSES.

We are officially informed by the Secretary of State for War that the following nurses will embark for South Africa on Saturday next: Superintendent S. E. Webb, of the Army Nursing Service, and Nursing Sisters E. M'C. Anderson, G. Beesley, J. M. Clay, E. A. Deacon, E. G. Eastmead, E. M. Edwards, A. M. Ferguson, R. Griffiths, E. M. B. Hall, M. Hilson, B. M. Hoare, M. N. Jephson, B. I. Jones, E. M. Pethick, S. T. Pollard, E. J. Storehouse, N. C. P. Tabeteau, M. E. Tippetts, and A. M. Winder, of the Army Nursing Reserve.

### THE PERSONNEL OF THE NURSES.

Miss S. E. Webb, the superintendent, has been a sister of the Army Nursing Service since May, 1886. She was trained at the Royal Southern Hospital, Liverpool, and was decorated with the Royal Red Cross in 1898. Miss Gertrude Beesley was trained, and has since been staff nurse at Swansea General Hospital; from October, 1896, to January, 1897, she was charge nurse at Brook Fever Hospital, and has since been attached to the Nurses' Co-operation. Miss Johanna M. Clay was trained at St. Bartholomew's Hospital, and has for some time held the post of sub-matron and superintendent midwife at the Northern Branch Hospital for Women, Brighton. Miss Brenda M. Hoare, who commenced nursing at a very early age, was engaged for two years at the Royal Alexandra Hospital for Sick Children, Rhyl. At the end of that time it was decided to pull the old hospital down, and Miss Hoare was offered the post of staff nurse at the Notts General Hospital. There she remained nearly a year, but finding that she could not gain an appointment as sister, owing to the fact that she had not entered as probationer, she entered in 1895 the Nightingale Training School at St. Thomas's Hospital and remained there for four years. One leaving she joined Miss Forrest's Co-operation for Private Nurses in Bournemouth, where she has been nursing since. Miss B. J. Jones was trained at St. Bartholomew's Hospital, and was subsequently staff nurse in the same institution. In November, 1894, she was appointed assistant matron to the Birmingham Infirmary, and since April, 1897, has been matron of the City of London Hospital for Diseases of the Chest, Victoria Park. Miss N. C. P. Tabeteau was trained at St. Batholomew's Hospital. She has been staff nurse at Chelsea Infirmary, and since October, 1897, has been engaged in private nursing. Particulars of the training and career of the other nurses are necessarily withheld, owing to the fact that they have not sent in their names for admission to "Burdett's Official Nursing Directory."

### NURSING ON THE HOSPITAL SHIP "SPARTAN."

Our correspondent on board the "Spartan" writes: "Again we have been to Durban, where we took on board a sad number of wounded who were brought by rail straight from the battlefield of Colenso. From three a.m., when they came on board, we worked incessantly. There were so many operations to be performed, the most numerous being the removal of bullets. Some came out clean like the kernel out of a nut, others were jagged and twisted, having torn and wounded the surrounding flesh. In one case the bullet entered below the eye; it travelled down, causing an ugly wound in the back, where it came out after having injured the spine, causing paralysis from the waist downwards. Some recoveries have been wonderful. In one case of fractured base the patient was two days comatose; several pieces of bone were removed; in five days he took interest in his nourishment, and is now apparently getting on well. Once, whilst a surgeon was doing his dressing, the sister holding the head, the ship suddenly gave a great lurch, sending trays, lotions, &c., flying, the cot swinging heavily against the surgeon on the other side. Luckily this happened towards the end of the dressing. When the patients arrived they were in a pitiable state; their erstwhile smart uniforms were in dirty rags; many men had not had their boots and socks off for two or three weeks, having had to live on the *qui vive*. Most of them had lost all their kit. One officer showed his silver cigar-case jokingly as being his 'whole luggage.' Oh! how they enjoyed their first meal on board, and how merry many were, in spite of their wounds! At the Cape most of the patients had to be landed in blue clothing, the orderlies fetching it back as soon as others had been procured, for that in which they had arrived on board was not fit to put on. Under the circumstances we could not refuse to let the officers have the last of our stock of slippers given to us by a patriotic fund, who also gave us dressing-gowns for the officers' ward, which are most useful. We are well supplied with games and books."

### COLONEL RHODES AS A NURSE.

The presence of amateur nurses at Ladysmith has already been explained. The investment of the town obviously prevented any reinforcements of trained nurses, English or Colonial. A correspondent who has lately returned "from the spot" gives us the interesting information that "men of gentle rank, anxious to employ a time of enforced idleness, and, above all, to help where help is urgent, are performing nurses' duties with most excellent results. Amongst these figures very considerably Colonel Rhodes (brother of Mr. Cecil Rhodes), who shares day and night work as though trained for the nursing profession. In lifting, carrying, and supplying good nerve when surgical deeds are necessary, Colonel Rhodes and his colleagues may be pictured as acting up to the highest expectations one likes to form of ideal nursing at critical times."

### AUSTRALIAN NURSES FOR SOUTH AFRICA.

One of the best answers to the groundless assertion that the claims of colonial trained nurses have been neglected is the fact that 14 nurses from Australia are actually being sent to South Africa under the auspices of the Colonial Government. We are able, through the courtesy of Mr. John Plummer, of Sydney, to give their names and other interesting details. The Australian contingent of nurses are under the superintendence of Miss Ellen Julia Gould, an English lady, who arrived in the colony in 1885, and has since devoted herself to hospital work. With Miss Gould is associated Miss Julia Bligh Johnston, who, during an outbreak of small-pox in Tasmania, was isolated with a number of other nurses amongst the patients. She holds the position of senior sister at the Sydney Hospital, is a member of the Royal British Nursing Association, also of the Australian Nursing Association, in the council of which she holds a seat. Of the remaining twelve nurses Miss Therese Emilie Woodward, a native of Sydney, commenced her career in the Hobart Hospital, subsequently going to England and gaining experience in the London Hospital, afterwards in St. Luke's Hospital, New York, and finally in the Royal Military Hospital at Netley, and the Herbert Military Hospital at Woolwich. The others, Misses A. Bessie Pocock, Nancy Newton, Elizabeth Nixon, Emily Hoadley, Penelope Frater, Elizabeth Lister, Austin, Marion Phillips Martin, Elizabeth Steel, Anna Jane Matchett, and Anna Gardiner Garden, each possess considerable experience in nursing work, and in volunteering for service in South Africa have, in more than one instance, made heavy sacrifices. It has been arranged that each of the nurses returning after the conclusion of the war shall, if she desires, be reinstated in her old position.

### THE INDIAN ARMY NURSING SERVICE AND THE WAR.

It appears from a correspondent, who writes from Umballa, that some of the sisters of the I.A.N.S. are indignant that they have not been sent to South Africa with the Indian contingent, especially as they consider that they have enjoyed exceptional opportunities of becoming acquainted with army nursing. A hope has been expressed that hereafter there will be a fusion of the Indian and the Imperial nursing services. It is pointed out that while nurses of the latter undergo six months training in the special routine of military nursing at Netley, the former are sent out to India without it, and are suddenly pitchforked into the nursing of tropical diseases. "Again," says our correspondent, "it frequently happens that a nurse in the I.A.N.S. is unable to stand the strain of continual Indian service. In existing circumstances, she is obliged to leave the service, whereas, in the event of a fusion, she might simply be transferred for a time to the home establishment. It must be confessed that the present system of having one sisterhood nursing the sick soldier in Malta and another in India is a curious anomaly."

### THE NECESSARY COMB.

An Army Reserve Sister writes us by this mail: "Land at last! I, for one, was really rejoiced, after having influenza on the voyage out. Influenza is bad enough within the four solid walls of a London hospital, but on board ship—it is past words to describe. The rocking and rolling of the vessel, the nausea, the stuffy cabin, and racking headache; but now that I feel so much better the unpleasant recollections are fading gradually like a bad dream scattered by the sunlight. The pleasure of seeing land again was great; this land is so beautiful. We got up early in the morning as we drew near the shore and peeped out of our portholes, to see the Table Mountain rugged and hazy, and Cape Town itself looking flat and uninteresting. At half-past eight a.m. we had breakfast and were all wondering what was going to become of us, for, as you know, we were told nothing more than that our passage was taken to Cape Town. A medical officer came on board, inspected us all, and sent nine of us to the Grand Hotel to wait for orders. At present I am a lady at large, but may be sent for at any moment to fill a vacancy. The first bit of business we did was a little shopping. The shops are large and well ventilated. Just fancy tea at 6s. a pound. Surely people in London have much to be thankful for. The flowers, however, are lovely. If I could only get someone to come with me I should like nothing better than to get right away up on the hills and pick some. I hear that up country a fine tooth-comb is an absolute necessity, so you can imagine the treat in store for us further on when it comes to wrestling with 'Africa's millions'; but nothing can make me anything but most delighted that I have come out."

### AN ACCEPTABLE GIFT.

The matron of the Samaritan Free Hospital, Marylebone Road, has devised and prepared a most acceptable gift to the wounded soldiers at De Aar Hospital. The sympathies of the patients and nurses of this institution have been warmly stirred by the sufferings of the soldiers, and knowing the need of pillows and cushions they resolved to help to provide them. Writing to Mr. Nightingale, of Dean Street, Soho, the matron asked if he would supply some filling for the pillows, and he immediately sent in 100 pounds of flock. Messrs. Spencer, Turner, and Co., Lisson Grove, W., promptly replied to a similar request for Turkey twill by presenting 100 yards for the removable covers. Zealous hands transformed these offerings into 70 pillows, which will this week be shipped direct to De Aar. A pocket has been placed in a corner of each case, and in each pocket a handkerchief. Between the outer and inner cases periodical literature has been put, so that each patient well enough to enjoy his pillow will find himself supplied with a pocket handkerchief and a book.

### A NEW SCHOOL FOR NURSES IN PARIS.

We hear that a school for the training of a superior class of nurses has been opened in Paris, to be called École Professionnelle des Infirmières (à domicile). It is the beginning of a movement which is sure to spread, till every hospital in the city is at last supplied with a band of gentle and competent women, trusted by the doctors, and blessed by the patients. In the meantime this school is not in a hospital, but in a sheltered corner of a lordly mans.on, built in the reign of Louis XIII., and at first the probationers will only be trained for private nursing. Candidates must be French, and if not possessed of a diploma for general education will be obliged to pass an examination. Sound health is, of course, a necessary stipulation, and the age of admission will be over 18 and under 25. The morning will be entirely spent in hospital, and the afternoon devoted to theoretic study. No less than six eminent doctors and surgeons of hospitals have already opened classes at the institution, and are giving their services gratuitously. Pupils are bound for two years, and pay 80 francs a month for board and training. At the end of the first year they will be allowed to choose which branch of profession they will pursue, medicine, surgery, or midwifery. They must then promise to remain at the institution for five years after receiving their diploma, and go out to nurse wherever they may be sent. The minimum salary is fixed at £60 a year. It is intended also to give gratuitous nursing to the poor. The inauguration of this beneficent work was held a few evenings ago in the Salle d'Horticulture, which was filled to overflowing by an appreciative audience. Dr. Paul Reclus, member of the Académie of Medicine, presided, and gave an eloquent address on the surgery of the past and the present, interspersing the account with many racy anecdotes as he went along. The speaker of the evening was M. Duclaux, member of the Institute of France, and director of the Institute Pasteur, who walked about all the time, as is his wont. His humorous and special pleading for the necessity of such a work carried the house by storm. The fairy godmother, who is president, and by her generosity has floated the good ship, is a Jewish lady, and a large proportion of the subscribers are of the same faith; of Protestants, also, there are not a few. The first candidate enrolled is the daughter of a pastor, and the next that of an advocate. The introduction of this system is no doubt one ste pin the direction of improving the condition of nursing in Paris; but we should like to have some assurance that the training these nurses receive in the hospitals is of a really practical character.

### THE CLERGY AND THE AGE LIMIT.

It cannot be the desire of the clergy of the Church of England to render it more difficult for educated women to earn a living. We find it, therefore, difficult to believe that the popular vicar of a large East End parish, whose name appears in an advertisement for a matron with a three years' certificate from a general hospital, and experience in ward management and housekeeping, can have seriously considered the conditions of age which he imposes. As a correspondent in our last issue points out, "from 27 to 32" are unreasonable limits. Herself a matron, she justly contends that to have passed through the three years' curriculum and the supplementary experience required at 27 is barely possible. As to the exclusion of all women over 32, it is impossible to condemn it too severely. The age limit, so often, though not, we are glad to know, universally enforced, is already harsh enough in its operation. Those who wilfully endeavour to reduce it are absolutely guilty of cruelty to women workers; and it should not only be the duty, but the pleasure, of teachers and preachers of Christianity to do everything in their power, both by precept and by example, to diminish the difficulties from which they suffer.

### NEW QUARTERS AT THE CITY LYING-IN HOSPITAL.

A new and convenient home for the pupil nurses at the City Lying-in Hospital has just been erected. On the ground floor is a large, pleasant sitting-room with eight cubicles. These have been apportioned to the night nurses, as they are quiet and dark. The upper storeys are divided into cubicles. Each floor has a new sanitary bath, a set of lavatory basins, and other conveniences. The cubicles are a fair size; they are divided by high partitions, and are to all intents and purposes private rooms. The furnishing promises to be adequate and complete. Considerable attention has been devoted to the ventilation, and the whole of the house is warmed by radiators. Much trouble will be saved by the telephone communication which has been established between the labour ward and the new home.

### SHORT ITEMS.

The Duchess of Roxburghe has written to Mrs. Adair, of New York, who is collecting money for the expenses of the hospital ship "Maine," that she had read both her circulars to the Queen, who was much interested and pleased, and begged the Duchess to express to Mrs. Adair her high appreciation of all that has been done through the kindness and generosity of the Americans, by which she is deeply touched.—At the annual meeting of the governors of the Birmingham and District Training Home for Nurses last Friday the chairman expressed his pleasure at seeing that provision is made for the old nurses, three of whom are already in possession of pensions. The salaries of the nurses have also been raised, and the institution is altogether in a satisfactory position.—Miss R. M. Bullock, of the Army Nursing Reserve, who sailed in the "Dunvegan Castle" for South Africa on Saturday, February 17th, was trained at the Norfolk and Norwich Hospital. For the last three years she has been a member of the Nurses' Co-operation. In 1897 she was awarded the silver medal of the Maidstone typhoid epidemic.—Miss R. J. Briggs, who sailed last Saturday, was also trained at the Norfolk and Norwich Hospital, and has since been staff nurse and sister.—Lord Roberts, in an official despatch from Paardeberg, states that there are 45 nurses at Kimberley, and that the wounded at the hospital are doing well. Subsequently the Commander-in-Chief expressed his intention to send as many wounded men as possible to Kimberley to be nursed.—The twelfth annual meeting of the Invalid Children's Aid Association will take place, by kind permission of the Duke of Westminster, at Grosvenor House, on Thursday, March 15th, at half-past three. The Marquis of Lorne will preside.

### Lectures on Medicine to Nurses.

By H. A. Latimer, M.D. (Dunelm), M.R.C.S. Eng., L.S.A.Lond.; Consulting Surgeon, Swansea Hospital; Past President of the South Wales and Monmouthshire Branch of Brit. Med. Assoc.; Past President of the Swansea Medical Society; Hon. Life Member of the St. John Ambulance Association, &c.

### LECTURE II.

### Some Causes of Disease—Nerve Force.

With respect to the modes of living which conduce to health they may be briefly summed up in the practical adoption of the maxim "Be moderate in all things." It is excess which ruins us—excess in work, in pleasure, in the indulgence of the appetites. So convinced of this are many folk that they would have us absolutely shun the use of things which, when taken in moderation, are undoubtedly of service, but which when abused are fearful agents of mischief. Foremost amongst such evils is the abuse of alcohol, an abuse which is so common in our country that a great deal of prevalent disease can be directly traced to it. I say this to nurses concerning the administration of alcoholic drinks to patients: Never give them on your own responsibility; only administer them as you would drugs on the order of the attending medical man. I am sure that people who have been cured of the habit of drunkenness sometimes relapse into it after illness through having been ordered to drink wines and spirits while unwell; for instances of this sort have come under my personal observation. Remember the well-known failing of such people. Many of them can abstain from drinking if the appetite for stimulants is unawakened, but their return to habits of excess often dates from the simple breaking of complete abstinence. Such an one is only too glad to be able to say that he has been ordered to drink on medical authority. Apart from illnesses which are self-induced disease often comes from the unsanitary houses we dwell in, or the unsanitary work in which we are engaged. In this last respect I consider a nurse is badly placed unless she will take extreme care to see that she gets proper daily exercise out of the sick-room and in the open air, and that she is exceedingly careful in her habits of personal cleanliness. When attending infectious cases she should see that all details of antisepticism are carefully carried out, and while she is securing this matter for the patient she should also be careful of herself. She is more exposed to risks of this sort than the medical attendant is, for she is constantly in the sick-room in close attendance on the invalid, while the former is there for only a short time. Familiarity with a danger is apt to breed carelessness, and one cannot but see and feel that many of the sad cases of nurses contracting enteric fever, for instance, are due to their not taking necessary precautions against this risk by applying the knowledge concerning infection which they have been taught when learning their profession.

In order that good health may be enjoyed it is necessary that we should obtain a full and sufficient amount of sleep by which to recuperate our vital forces. What this amount is varies very greatly in different people, for whilst some can enjoy comfort and good health with but four or five hours of it in the twenty-four hours, there are many—and these are certainly in the majority—who cannot do well on less than eight of repose. Habit plays a great part in the matter; but it is likely, in addition to this, that the benefit we receive from sleep depends largely upon its intensity as well as its duration. General sleep is made up of individual sleeps, and the more completely all our organs are resting the more completely is the central nervous system in full repose.

In the hurry and scurry of modern life the thinking portions of the brain are so often over-excited and overworked that sleep is banished, and it is quite astonishing to see what a number of people form the habit of taking drugs to procure it. Nothing can be more pernicious than this custom. Almost all the sleep-inducing drugs are harmful to the system when taken habitually, and yet this habitual use of them is what is established in the great number of cases of people who resort to their use. I advise you to shun such drug-induced sleep. If you once take to the use of morphia, sulphonal, or any such drugs, you run the greatest risk of becoming an habitual abuser of them, and then good bye to the full enjoyment of life.

In connection with this question of overworked faculties it is curious to note how unwilling the great majority of people are to recognise the fact that it is not hard work which injures them, but continuous work of the same parts. You may tax the brain all day long if you will employ different parts of it. Many of the longest-lived people of eminence who have been brain-workers have been noted for their persistent energy, but if you will observe what they have done you will see that they varied their labours, and avoided "harping on one string." You will find such people distinguished in several directions, being, for instance, politicians, scholars, and novelists at the same time. It is your lazy people who contrive to do nothing, and who tell you that they have not time enough to spare for any work they are asked to do. It is now proved that the brain contains various "centres" for controlling movements in the body, and it is certain that it also contains centres for intellectual work. Call upon these centres for work at different times and you give not to those ones which are not so engaged but call persistently upon the same ones day after day and you exhaust these to whom you have given no rest. This would appear to be particularly the case in mental work involving money speculations. How often do we see the business man engaged in the engrossing occupation of mere money-getting break down at middle age from some brain disease? Here, as a rule, there is no variety of thought. Morn, noon, and night the mind is engaged at the same problems, and one cannot doubt but that the same set of cells in the brain are being used in the work.

The subject is a doubly important one when we consider that the brain dominates the body, and that without healthy nerve-force the various organs within us cannot carry on their functions correctly. A practice extending over many years, and affording considerable experience in people and disease, has convinced me that many of the illnesses which are called "functional" are due to exhaustion of the nervous system; and when one reflects upon the *rôle* played by this part of our economy it is not difficult to see how perturbations in it will cause disturbance of function throughout the body. No organ can do its work unless properly stimulated by the nerves which supply it with nerve force. Hence it results that many cases of indigestion, disturbance of the heart's action, and so on, are due entirely to disturbances in the flow of this vital fluid. If one wants to study an example of this fact it suffices to observe a sufferer from an acute hysterical attack, for here we find an exhibition of nerve-disturbance in its most violent form, and, at the same time, of a very ephemeral duration. With, in many cases, great suddenness the voluntary muscles will be endowed with a degree of power which they never attain to under ordinary circumstances, the breathing will be quickened, and the intestines will be distended with large volumes of gas, and at the termination of the fit the bladder will be actively engaged if expelling great quantities of a clear, limpid urine which has been quite suddenly secreted by the kidneys. In these hysterical conditions we see the results of increased and on retarded supply of nerve force contrasted in the same patient; for whilst on the one hand she exhibits phenomena of force such as I have been describing; on the other hand, her will-power is in abeyance, and she lacks the force to perform acts which she would like to undertake.

### Arrival of the "Princess of Wales" Hospital Ship at Southampton.

### THE SICK AND WOUNDED SOLDIERS ON THE VOYAGE HOME.

### THE ROYAL VISIT.

A nurse on board the "Princess of Wales" Hospital Ship, which arrived at Southampton on Sunday, sends us the following:—

"We started on January 31st. Seventy-five men were embarked in about two hours and a half, looking sad and careworn as they came on. Most of them were able to walk with assistance. One man of the Black Watch was carried on, being unable to stand; he was wounded at Magersfontein. When we arrived he was able to hop with a stick into Netley town. All the soldiers look much better for the voyage. Some of them did not like the swell we experienced at Madeira, and retired to bed; but they were the exceptions. Smoking was allowed after half-past five a.m. on deck, and 'Tommy' took full advantage of the privilege. with the Prince came on Monday and went round. The men were at dinner. Their Royal Highnesses seemed much pleased with the arrangements, and were greatly interested in hearing about the men. When the latter stood up the Princess, in her usual gracious way, said: 'Sit down; don't tire yourselves.' One man was very proud because, as he said, the Princess talked to him most. She went to the train amid much cheering from the orderlies and crew. The sun, which was shining brightly, showed off the flags to great advantage, and made one feel proud of belonging to the vessel. After the Royal train left the men changed their kit, drank tea in the dock provided by the 'Absent-minded Beggars' Fund,' and were quickly entrained for Netley."

By kind permission of the Proprietors of the *Daily Graphic*.]

The Alexandra Ward of the "Princess of Wales" Hospital Ship.

Those who were well enough also enjoyed the large sail bath which was filled every morning for their benefit. Great care was taken about time, and as the weather got colder the mornings and evenings on deck were shortened, and poor 'Tommy' had to defer his early pipe a few hours, and amuse himself in the evening with games in the wards. We had a very exciting concert, given by the 'Tommies,' the crew, and the orderlies. One man recited a very pathetic piece which he had written about Magersfontein. The Queen's chocolate, which they all had, was greatly treasured. Some of them allowed us a taste; others kept it as a family heirloom. One man who was shot in the back, and was found some hours after partly conscious, was quite unable to walk when he came on the ship, but he is able to get about well now, and is very amusing. We reached Southampton on Sunday morning, and received a warm welcome from the ships we passed. The Princess of Wales

It may be added that, though the proceedings on the occasion of the visit of the Prince and Princess of Wales were private, the Prince and Princess and the Duke of York went through every ward and down into the troop decks. Major Morgan escorted them round and explained the nature of the wound from which every man had suffered There was no speech-making, but there is not one of the 176 men on board the "Princess of Wales" who cannot boast that the wife of the Prince of Wales has spoken to him words of comfort and encouragement. The description of the sufferings of some of them so touched the Princess that she repeatedly exclaimed, in the course of her tour, "Oh, this terrible war; this horrible war!" The Royal party expressed their opinion that the state in which the men arrived was highly creditable to Major Morgan, to Miss Chadwick, the superintendent nursing sister, and to the nurses, female and male, who have been in charge of them.

### The Economics of Good Nursing.

We are so much in the habit of looking at nursing from the purely humanitarian standpoint, that we are apt to forget that it may have a justification from another point of view. Pity for the suffering is indeed reason enough for our wishing that those who nurse the sick should be trained for their work, and that the employment of such trained nurses should become more and more general; but there will always be selfish and hard-hearted souls who are not sufficiently moved by the thought of others to make them desire to see the services of the nurse provided even for those who cannot afford to pay for her help. But a wider outlook will show that in the complex machine of modern society, where each depends on the work of all, it is each man's interest that all workers should be well nursed in sickness, so that they may be soon restored to health.

### The Value of Efficent Labour.

Every year the value of labour depends more and more on its efficiency. The work of the poor, ill-fed, ill-housed, ill-paid labourer becomes more worthless every year. He is easily tired, he is unintelligent, he makes stupid mistakes from sheer ignorance. Just because the modern workman has to do with costly machinery where his forefather had to do with simple tools, his stupidity is more mischievous, more costly to his employer than his forefather's blunders. Unless he is worth a great deal, he is worth less than nothing to society; he is a burden and an encumbrance. This is the best justification for using some of the profits of capital in improving the dwellings, the education, the whole environment of the working classes. Let no man say that the money so expended is "conscience money." The man who has built up a great industry in any place is a benefactor to that place. He has, in very fact, "made two blades of corn grow where but one grew before," and he has a right to the gain that his wisdom and energy have earned. But in so far as he works by instruments which he directs and controls, it is to his interest that these instruments should be the best of their kind, and in perfect condition. Now many of the instruments he employs are human beings, the most difficult of all machines to keep in working order, all the more difficult that they have a volition of their own, which does not always lead them to do the best for themselves, from an employer's point of view. Any employer of experience will tell you that it is best for him that his workers should be in good health. A bad physical condition means a bad mental condition, from which anything may result. The evils that have been wrought even by such a common ill as indigestion have been chronicled before now. Is it not said that the first battle lost by Napoleon, the event that first turned his fortunes downwards, was lost through the great general having indiscreetly dined off some indigestible fare, and not having mind and temper under such perfect control as usual? Lesser Napoleons suffer in the same way every day, and in their own sphere the results are just as disastrous.

### The Question of Health.

If then it is the employer's interest to get as much work of as good quality as possible out of his workers, it is his interest to keep them in health. In the past employers have not always seen this. When men and women were kept at work till they were so tired that they could scarcely stand, and their weary eyelids fell over their eyes, the plea offered by their masters was that "the last hour's work gave the profits." When hours were shortened by law, the masters found, to their surprise, that profits increased rather than diminished. As it was put, they found that it was in the last hour's work that their profits had been lost. The increased vigour, earnestness, and watchfulness of the workers made up, and more than made up, for the shorter time they were at work. And this has held good of every improvement in the condition of the people. What employers have given with the one hand they have received back with interest with the other. Short hours, good houses, big wages, when these are wisely spent, have all profited the donors as well as the recipients.

And it is the same with health. It is a wise employer's aim to prevent sickness in those he employs, and if in spite of him it comes, it is as decidedly his interest to restore health. Quite apart from any questions of compensation, if his workers are injured in the course of their work it pays him to have a man whom he is going to employ again made well as soon and as thoroughly as possible. It is his interest that the workman should be attended by a good doctor, and it is also his interest that he should be well nursed. What an employer of labour subscribes towards the maintenance of a district nurse he might reasonably regard as invested in an insurance fund for maintaining his workers in a state of efficiency.

### The Nurses and the Workers' Family.

It may be said that though this may hold true of those whom he actually employs, he derives no benefit from the nurse's attendance on members of the worker's family—the wife and children who are as likely to require her services as the man himself. But is it unreasonable to say that when there is sickness in the home, the average man, be he rich or poor, master or man, is not himself? He cannot concentrate his mind upon his work as he does when all is well with those whom he loves. He may get through his work after a fashion, and, of course, there are employments where less care and attention will suffice to produce a passable result than others, where a man's mental condition matters comparatively less; but there is none where there is no difference between a good worker and a bad one. And a bad one the average man is bound to be when he has anxieties outside his work at the moment. And it is hardly necessary to add that in the homes of the humble sickness obtrudes itself more upon the family comfort than it does among the rich. The sick one's moan or restlessness disturbs the whole family. And when it is the wife who is ill it is inevitable that household affairs should be neglected. The comfort and well-being of the breadwinner suffers, and therewith his efficiency as a worker. This is the point we wish to emphasise, not to the exclusion of other and indeed higher considerations that bid us extend the opportunity of good nursing to the poor, but only to show that, even on economic grounds, the nurse, carrying healing and comfort with her, is deserving of the support of all.

### Novelties for Nurses.

### LADIES' BICYCLES.

(Anderson, Anderson, and Anderson, 35, St. Paul's Churchyard, London, E.C.)

We have carefully examined one of the above firm's special ladies' bicycles. It is called the "'Varsity," and is listed at the popular price of ten guineas. It is a wonderfully well-finished machine at the price, with double-plated fork ends, mud guards with plated stays, spoon brake, celluloid and leather gear-case, and ½-in. roller chain. The rims, which are of the Westwood variety, are fitted with Dunlop Multiplex pneumatic tyres. In fact, in most of the essential details the parts are such as belong to a high-grade machine listed at a very much higher price. The machine is just such as would commend itself to a visiting or district nurse. Those who are on the look-out for a machine should write to Messrs. Anderson, Anderson, and Anderson, at 35, St. Paul's Churchyard, for lists and particulars.

### Hospital Sketches.

### THE STEWARD'S STORY,

Agnes Ellwood was a pretty girl of twenty-two. She had a pleasing manner, and, for one of her station, a refined and winsome tongue. Her home was with her grandmother, who lived off the Marylebone Road. Agnes had been left an orphan when an infant, and her old grandmother, whose fixed income was only fourteen shillings a week, found it necessary, as she became more advanced in years, to relinquish some of the needlework that she did at home and to allow the girl to go out into the world and bring in the few extra shillings that were essential, if comfortable shelter and enough food were to be had for them both. At seventeen, therefore, Agnes had gone as junior assistant in an Edgware Road toy shop. She remained there for three years, when she had an illness and resigned her post. On recovery she sought a new situation, and was successful in obtaining a place as assistant-in-charge of a small but high-class confectionery business in a good part of Oxford Street.

One day, after two years' conscientious attention to her employer's interests, she fell from the top of a high step-ladder to the floor of the shop. She had been reaching a box from the topmost shelf, and had overbalanced herself. At the moment of the occurrence the shop happened to be empty, for the younger girl, who was stationed there as her helper, was out at dinner, and no customer was about.

She was lying on the floor of the shop behind the counter, groaning pitiably, when discovered by a kindly customer, who immediately summoned a constable. He, good man, procured some brandy, and presently had the satisfaction of seeing the eyes of the injured girl open and light up with intelligence.

"Oh, my leg, my leg, it is broken," were her first words. The kindly customer, who had remained in the shop, hailed a passing cabman, and, acting on the instructions of the constable, requested that an ambulance might be fetched with all possible speed. This was done, and Agnes, a few hours later, was lying with closed eyes and calm brow in number fourteen bed in Victoria Ward of the Royal Central West Hospital.

Her aged grandmother had been apprised of the mishap, and had just departed from the ward after a brief visit.

Before going off duty the Sister drew near to Agnes's bedside and tenderly inquired whether she could do anything more for her, and Agnes replied—

"Would you do me a *great* favour if I made a *great* confession?"

"If it is possible, I will," whispered the Sister in reply.

The patients in beds number thirteen and fifteen were sleeping peacefully, and the long, dimly-lit ward was hushed in silence, save for the occasional soft rattle-rattle of the night nurse's chatelaine as she went quietly from bed to bed to see that all was well, as was her wont on taking over her work.

"Well," began Agnes, looking up with perfect trust into the Sister's kind face, "I have been deceiving my grandmother, my only relative, for a long time. I have a friend —he is a gentleman—who sees me regularly, and who has been goodness itself to me for a whole year, but whose real name I don't even know."

"Really, my dear," soothingly began Sister, thinking that after all the patient was about to divulge an all too frequent tale of woe, "is it wise to tell me of this! Why not wait till morning and think it over, and then perhaps you will have decided to tell your grandmother, whose claims, I am sure, you ought to consider first."

"But I can't wait, and I don't want grandmother to know anything," answered Agnes, piteously. "There's nothing bad to tell; only I want him to know that I am here. He may not find out where I am, and I can't bear to think that he should worry about me."

"Does he not know where you live?" asked Sister.

"No; he's never asked. He always comes to see me at the shop," was the mystifying reply.

"Where does he live, then?" inquired Sister, sitting down on the stool at the patient's head, and beginning to feel a deepening interest in Agnes's disclosure.

"I don't know where he lives, but I write to him at an office in Piccadilly. He told me I might send letters for him there; and I once went there, hoping to see him, and found it was only a place that they call a 'bureau,'" said Agnes.

"I see," said Sister, meditatively; "but you must know his name, else how do you write to him?"

"I know him as Mr. Richard Corner, but that isn't his real name, because I once picked up a letter that he let drop, and it was addressed quite differently—to some road in Kensington, I think. That was during our early acquaintance, though, and I did not take any particular notice of it. You see, I didn't know then that I should learn to care for him as I do," answered Agnes faintly.

"Does he know now that you care for him?" questioned Sister.

"Oh, yes; I feel sure that he does. Why, I call him 'Dick,' and I see him several times every week. I've been to Richmond, and Windsor, and Hurlingham with him for a whole day, and often to the theatre. He's not at all like any other of the men who come to the shop. He's such a gentleman. I would trust him under any conditions."

"He's never attempted any liberty, then?" said Sister, with reassurance.

"Never," was Agnes's emphatic response.

"But don't you know anything about him privately? Who is he? What does he do?" asked Sister.

"I don't know who he really is, and I don't try to find out. I'm certain he's a good man, and that he gets his living honourably. He doesn't work with his hands, I can tell that, and he calls at the shop at all times of the day, so I think he must be his own master," replied Agnes in tones of conviction.

"What an odd affair," murmured Sister, more to herself than to her pretty patient. "And how do you propose to act now?"

"I want you to write to him for me and tell him I am here and that I want to see him so much," answered Agnes beseechingly.

Sister promised to think about it. Though she pointed out that it was not a right thing to do without first informing the old grandmother.

"But I am over age, and I keep myself," protested Agnes. "I don't tell grandmother, simply because she likes to think that she has all my love. And I'm sure when you see him you'll be satisfied."

After much feminine deliberation Sister did write a few lines in the third person, posting the note late the same night.

Towards noon on the following day a letter arrived at the hospital from a firm of solicitors in Ely Place. It was addressed to "The Governor," and in the absence of the secretary the steward opened it.

"Sir,—We are instructed by a client who is interested in a patient named Agnes Ellwood, now lying in your hospital, to ask that you will favour him with a report as to the exact nature of her injuries, and that you will further be good enough to say how the patient is progressing at the present time. A cheque value ten guineas is enclosed herewith, which we have been desired to draw and forward to you as a donation to the funds of the hospital."

I saw the Sister, and I saw Agnes. The former was quite sure that the letter I had received was the direct result of her note of the previous night. Of course, we kept Agnes in ignorance of it, for it seemed pretty clear that Mr. Richard Corner was a wily fellow and did not intend to reveal his identity unless he himself was readily disposed to do so.

A letter, containing full information on the questions raised, an acknowledgment of the donation, and a visitor's card, was promptly despatched, and I awaited the outcome with some little curiosity.

My office is locked up between five and six every afternoon, but that action by no means signifies that work ceases. The door of my private room is assailed times without number by sisters and nurses and porters and servants who wish for this and want that right up to midnight, if I happen to remain for the whole of the time on the premises. At nine o'clock on the evening following the receipt of the solicitor's letter respecting Agnes, Brown, the night porter, knocked at my room door and announced that a gentleman was waiting at the entrance in his brougham. He had sent up his card and could I see him.

I glanced at the card and Brown must have noticed my absurdly emotional jump. "Yes, I'll see the gentleman, Brown. Show him up at once," I said, reading the few words printed on the pasteboard again—the name and address of an eminent dramatist.

"I'm exceedingly sorry to trouble you at this time," said Mr. ——, entering my humble apartment, "but I've come on a decision arrived at only an hour ago, and the strangeness of my errand will perhaps account for my late call. You wrote yesterday to my solicitors concerning a patient you have here named Agnes Ellwood."

"We did," I assented.

"I am the client of theirs who desired information, and I've come here now to say that I really cannot see the girl. I made her acquaintance in a curious way. She keeps a sweet-stuff shop in Oxford Street, where I used to make small purchases for my numerous little friends. Her face attracted me strongly. I chatted with her and found her intelligent and winning. When I first spoke to her I was rehearsing one of my plays at a West End theatre. I was not pleased with the representation of one of the characters, and I more than once thought seriously of offering to Agnes an opening on the stage that might have proved to be the first step of a brilliant career. But I reflected, and in the meantime I grew to like her. Finally I determined not to mention the stage to her, for its temptations are not such as can be resisted by everyone, and I knew her to have always been noble and good. I gave her a name other than my own. I walked out with her occasionally, and I took her little driving excursions into the country. As a married man I suppose my conduct was open to censure, but that I don't care a hang about, so long as I have not irreparably hurt her. The little romance has given me many hours of innocent happiness, and I think she has also found pleasure in what I have been able to let her share. She has not had a single present from me, and promises on all subjects have been absent from all our talks."

I moved uneasily in my chair. I felt uncomfortable. Why should this famous man make a confidant of me?

"But," he resumed quickly, and ignoring my movement, "I want to do something for her now, and I want you to help me. I will set her up in business for herself, or do anything in reason she wishes, but I really cannot see her again—for her own sake; I begin to see that it will not do. I did think that all shopgirls have flirtations, and that in many cases they end without calamity; but Agnes is not an ordinary shop-girl, and I ought not to have done what I have."

I accepted a fine-flavoured cigar from my visitor, promised him my secret assistance, and bowed him out.

Agnes was skilfully approached by the Sister on the subject of Mr. ——'s offer, but all to no purpose.

"He won't see me; then I want nothing else," she said simply.

It seemed that all night long Agnes had lain awake anxiously thinking over her position, and had arrived at the wise conclusion that she had done wrong in allowing herself to be drawn into a secret friendship with one of whom she had learnt nothing more than that he was wealthy and generous. The error of her way was now perfectly clear to her, and with a happy, albeit tear-stained face, she told Sister that she had resolved not only to accept nothing, but never again to see her former patron-lover.

"I'll get a new situation, and start afresh; and perhaps some day—some day——"

"Well," exclaimed Sister, coming to the rescue, "perhaps some day you will meet someone else who will love you enough to give you his name and address, and make you the happy wife that you deserve to be."

### Presentations.

Park Hospital, Hither Green.—On Friday last the nursing and domestic staff of the Park Hospital, Hither Green, presented to their matron, Miss E. J. Atkins, a handsome dressing-bag with silver-mounted fittings, travelling rug, and a Russian leather writing case, on the occasion of her departure for service in South Africa. She takes with her the good wishes of all her staff, and many regrets that she is leaving.

The National Hospital for Consumption, Ireland.—As already announced, Miss J. Gertrude Powell, who has been lady superintendent at the National Hospital for Consumption for Ireland, Newcastle, co. Wicklow, since its opening in March, 1896, has been appointed lady superintendent to Mercer's Hospital, Dublin, and begins her duties this week, carrying with her the best wishes from all who know her. Miss Powell, by her devotion to duty and her unfailing kindliness, has, says a correspondent, won the affection and esteem of all who knew her. Desiring to express to her their goodwill and their own sense of loss at her departure, the medical and nursing staff of the hospital have presented her with a silver-mounted dressing-case, a silver preserve dish, and a pair of silver butter forks. She also received from all the patients a case of silver afternoon tea spoons, and from the servants a silver toast rack and butter cooler.

### Minor Appointments.

Stroud Hospital.—By a printer's error last week the appointment of Miss Kate Elliott as Sister was headed "Strand" Hospital.

Newcastle-on-Tyne Union Hospital.—Miss Theodora Stokle has been appointed Charge Nurse. She was trained at the same institution.

Peterborough Union Infirmary.—Miss Harriet Shaw has been appointed Assistant Nurse. She was trained by the Meath Workhouse Attendants' Association at the Home for Invalids, Aubert Park, Highbury.

Wandsworth and Clapham Union Infirmary.—Miss Ana Maria Riley has been appointed House Sister. She was trained at St. Bartholomew's Hospital, and her subsequent engagements have been at Kensington Infirmary, Colonial Hospital, Gibraltar, and the Nursing Home, Malta.

Hartlepools' Hospital.—Miss Lydia Ritson has been appointed Sister of the women and children's wards. She was trained at the Chesterfield Hospital, and has subsequently been staff nurse at the Royal Infirmary, Sheffield, and at the Kendal Hospital, and charge nurse at Hartlepools Hospital.

Lexden Union Workhouse, Stanway.—Miss Rosetta Norvall has been appointed Superintendent Nurse. She was trained at the Brownlow Hill Infirmary and Hampstead Infirmary under the Workhouse Infirmary Nursing Association, and has since been assistant nurse at Eastbourne Infirmary and Epsom Infirmary, and charge nurse at Portsea Island Workhouse Infirmary.

London Temperance Hospital.—Miss Florence Wooll has been appointed Night Superintendent. She was trained at Guy's Hospital for three years, and afterwards at the East London Hospital for Children at Shadwell for two years. She has since been on the staff of Guy's Hospital Nursing Institution; sister at the Royal Alexandra Hospital for Children, Brighton; and sister at the East Dulwich Infirmary.

### The Dublin Nurses en route to South Africa.

Miss Treacy, superintendent of the City of Dublin Nursing Institution, sends us the following letter which she has received from one of the Irish nurses who left Southampton on December 31st:—

Ss. "Moor," Lat. 20·59 N., Long. 17·40 W., Saturday, January 6th, 1900.

Dear ——,—The others have told you of our experiences at the commencement of our journey, so I now add my tale, and it has been a tale of woe for me until two days ago, when I came round a little. The ship has rolled the whole voyage. We were twenty-four hours late in arriving at Madeira. The others pulled me up somehow, and we went ashore over such transparent water—so blue, and such a summer sky! The view is a picture: town built on hills, the highest snow-topped. The climate felt like June. We had four hours ashore, so we got a guide to show us round. We went first to the English Hotel to inquire for Nurse ——, but she was not there. We pulled the most exquisite hot-house flowers by the roadside, such roses! And as for fruit; we got heaps of strawberries and other rare fruits, as much as we could carry away for a few pence. The streets are paved, and the conveyances are sleigh-like affairs drawn by little bullocks. The diving boys are great fun, and the natives very polite, but they thought that we were mad to go to Africa. The women were dressed in cotton gowns, Spanish fashion, and the girls' soft dark eyes charmed us as well as the graceful way they walked. The weather now is perfect summer. We had some music on deck after dinner, including "Killarney" as a cornet solo, and all we Irish sat beside the band and clapped it vigorously. There are splendid horses on board, and the owners are getting rather cross as the nurses are continually stuffing them with sugar. Colonel ——— is in charge of the eighteen nurses, and he has no idea where he is going, or any of the nurses. I hope you can decipher this, as the ship is rolling and creaking so much it is hard to balance one's hand, and I feel shaky still. Nurse ——— has a bad throat, and lost her voice the last two days. Nurse C. is well, and looks all the better for the trip; they were both extremely kind to me the week I was so ill. Our hats are the envy of the other nurses, they have sailor hats, which are heavy and uncomfortable, and do not keep off the sun.

Sunday, January 7th, 1900.

We had church in the saloon to-day, the captain read the service; all the officers, nurses, and sailors were in full uniform, we in our brown cloaks and bonnets. There was quite a strong choir of sailor boys in their blue jerseys and bare feet. This is the calmest day we have had, such lovely sunshine. We often say to each other "What kind of weather is it in Dublin?" We saw Cape Verde to-day, and whales playing about quite near us, and flying fish; we see phosphorous at night on the water. All the food served is very good, and we are so much waited upon that one fears one will almost forget how to do it for one's self by the time Capetown is reached. We were disappointed at not getting that proof of the photograph at Southampton. We hope you can send us your own as well with it. Miss ——, from Aldershot, is very nice. She is very pleased that we have only our uniforms with us; and she says that any who have brought a different dress for every day in the week are most unbusinesslike.

January 9th.

We had a funeral at sea to-day—the ship's cook, 22 years at sea; he died of influenza; one of the nursing sisters has been very bad with it, too. The man died at 2 o'clock and was buried at 6 p.m. The whole ceremony was most impressive: The ship's bell tolled, the ship stopped, and all officers and crew, in their best clothes, marched behind the stretcher, which was covered with the Union Jack. The captain read the service, and, by some pulley arrangement, the body was shot into the sea in almost less than a second. The heat now is intense; we crossed the line at 10.30 to-day. We have an artist for *Black and White* on board, and a war correspondent for the *Daily Mail*.

Sunday, January 14th, 1900.

1,223 from Table Bay. It is much cooler now, and the wind is very high. We are sorry that the journey is coming to an end. There are sports and amusements of different kinds on board, and a ball a few nights ago—quite a grand affair, the deck polished and the ball-room screened off with flags and plants; we got plenty of dancing. There was also a concert on board and a collection afterwards for the widow of the man who died. Nurse —— was specially asked to take the plate round, and she got £14 for the poor woman. Before posting this we will, if possible, tell you where we are to be stationed. We all hope you are well, and are always thinking of you all at the home.

### Appointments.

Private Hospital for Women, George Square, Edinburgh.—Miss Joey Forsyth has been appointed Matron. She was trained in Longmore Hospital for Incurables, Edinburgh, for two years; in Western Infirmary, Glasgow, for three years; was engaged in private nursing for a year, and at present acts as staff nurse in Longmore Hospital.

Wakefield Union Infirmary.—Miss Lois Lightowler has been appointed Superintendent. She was trained at the Fir Vale Infirmary, Sheffield, and afterwards became charge nurse at the same institution. She has also held the position of private nurse at the Huddersfield and Halifax Nursing Home; night superintendent of the Toxteth Park Workhouse Infirmary, Liverpool; and sister at the Wakefield Union Infirmary.

Preston Royal Infirmary.—Miss Grace Goffin has been appointed Assistant Matron. She was trained at the North Staffordshire Infirmary and Eye Hospital, and has since been assistant house matron of the Southport Convalescent Home, sister at the Royal Derbyshire Infirmary, and assistant house matron at the City of Manchester Isolation Hospital.

St. Pancras' Parish Schools.—Miss Annie Saunders has been appointed Assistant Matron. She was trained at the North-Eastern Hospital for Children, Hackney Road, and was out-patient sister in the same institution for two years. She was then on the private staff of the Hospital for Sick Children, Great Ormond Street, for a year, and has since been charge nurse at the Free Hospital for Sick Children at Nottingham.

National Hospital for Consumption, Ireland.—Miss Hoadley has been appointed Lady Superintendent. She was trained at Guy's Hospital, was matron of Goole Cottage Hospital, Yorks, for two years, and for the last year has been assistant matron superintendent at the Royal Infirmary, Preston, Lancashire. Miss Hoadley holds the silver medal for five years' service at Guy's Hospital.

### A Day in My District.

By a District Nurse in London.

It is half-past six in the morning, and there comes a smart rat-tat at my door. I arouse myself sufficiently to utter a sleepy "Thank you," and to turn on the other side, with the very pleasant consciousness that I may indulge in another half-hour's rest. At seven the various whistles and bells from the many factories which surround me on every side warn me that that last blissful half-hour is at an end, so after giving the subject a certain amount of serious thought and consideration (worthy of a better cause), I spring out of my cosy nest, light my oil stove, and set my kettle on to boil. I then proceed to dress in great haste, have my breakfast, and read. My next move is to start my household duties, all of which are performed in one room, and which I firmly believe take the longer time owing to the limited space. Having brought up my day's supply of water and coals I am ready to commence my day's work, but if there still remain a few minutes before nine o'clock I do a bit of sewing.

### The Morning Round.

At nine punctually I don bonnet and cloak, seize my bag, and march out to the scene of action. My first call is at the Mission Rooms to see if any fresh message awaits me, but as I have quite as much as I can do at present I am distinctly relieved if there is not. My first patient is a sweet little golden-haired girl suffering from pneumonia; but, sad to relate, her behaviour is very far from sweet from the moment I enter the room until I leave it again. I take temperature, pulse, and respiration, sponge her all over under a blanket in front of a blazing fire, make her bed, and apply fresh poultices, all of which occupies me very little under an hour. My next visit is to a woman, who besides having to be washed and have her bed made, has a very bad wound on one of her legs. To dress this and to arrange an improvised cradle takes some time. Almost before I have left the house she sends the whole arrangement flying to the other end of the room. I pass on to a man with chronic rheumatism and a bad bed-sore, caused by neglect on the part of friends, one of whom used to assist me at my task. Every now and then she would break in with some very consoling remark such as, "Yes, my dear, you are very bad," or "Poor dear, he can't last much longer now." Needless to say, I very soon discovered that I could manage quite well without her assistance, and gave a gentle hint to that effect, which I am glad to say was promptly taken. Then come short visits to two or three children in various stages of recovery from bronchitis, till those useful factory bells and the shouts of the children let loose out of school warn me that I must retrace my footsteps to the home of a little boy with hip disease, who by the aid of a Thomas' splint and a pair of crutches, wends his way to school every day regardless of wind, rain, or fogs. Therefore the only time when I can do his dressing is in his dinner hour.

### The Dinner Difficulty.

As soon as I leave my hip boy I usually return to my own dinner. Ah! the trouble and anxiety those dinners cause me, and how much I long to leave them out of my day's programme altogether, rather than have the trouble of getting them. Sometimes I leave a dainty little stew simmering on my stove thinking I have turned the light just nicely, to come in and find the wick has flared up in my absence, blackened everything in my pretty little room, and sent the stew itself gaily bubbling and dancing over stove, fender, and floor. Another time I return to find my food burnt to a cinder, or else, owing to the light being turned too low, it has gone out altogether, so that a tiny joint, supposed to be roasting in the oven, is found in the same condition as I left it. Sometimes—but here I draw a veil over further disclosures lest my readers should agree with my own conclusion that cookery is not my *forte*.

### Afternoon Visits.

I very often discover a message at my room, sent after my call at the Mission Rooms. Such is the case to-day, and I make my first afternoon visit to the address given, where I find a man with a very severe attack of pneumonia. He had been ill for some days before his wife could make up her mind to accept my help in nursing him, and she now informs me that "he hasn't had a wash for a week." I promptly reply that it is quite time for him to have another then, and if she will get me the hot water and clean linen we will at once proceed to make him a little cleaner and more comfortable. I am agreeably surprised to see with what alacrity she gets all that is wanted, rendering such prompt sensible assistance that I could at once see she only wanted to be told just what to do, and it would be done. When I come across intelligent help it quite cheers me, for some of these poor people have such a horror of soap and water and fresh air, audibly expressing their firm conviction that the patient will certainly die under such treatment. If one can overcome any of their prejudices, one feels that a great victory has been achieved.

### A Second Tour.

After leaving this case I make a second tour over my district, which, by the way, contains sixteen thousand inhabitants, and covers a considerable space of ground, and now I dress the few surgical cases which I have at present. These, I may say, vary considerably in number, size, and severity. At one time I have quite a number of children who have met with small accidents, such as cut and bruised heads, eyes, hands, and knees; at another time all the cases seem scalds and burns. I get back to tea, and being service night, when I always like to attend if possible, I go out as soon as I have finished to those cases requiring two visits daily. Then, after service, I go home for the night, but my work is not quite finished yet. There are dressings to be cut, a bag to be stocked, and perhaps a cotton-wool jacket or a particular kind of bandage to be made for use on the morrow. After that I have an hour's study before putting away all work to write letters or read. Then come supper and bed, but for the latter I have no stated time.

### District Nursing.

I am frequently asked which kind of nursing I prefer, hospital or district, to which I invariably give the same reply; I love both kinds, and each have a special advantage that the other cannot share. Of course, it is quite impossible in the homes of the poor (or the rich, either, sometimes) to get the order, method, and regularity of hospital life, not to mention the use of up-to-date appliances to aid the recovery, and to ensure the comfort, of our patients. But, on the other hand, by going in and out of their homes, day by day, we get to know our poorer brethren better, and can sometimes give them a few words to brighten and cheer their often dull and dreary lives; thus, as we go from house to house, trying feebly to follow in the footsteps of Him of whom it is said, "He went about doing good."

### Where to Go.

Dowdeswell Galleries.—An exhibition will be opened on March 5th of water-colour drawings of Italian cities and Swiss mountains by Harry Goodwin at these galleries, 160, New Bond Street, W.

### Echoes from the Outside World.

AN OPEN LETTER TO A HOSPITAL NURSE.

The excitement caused by the relief of Kimberley, although it was considerable, bore no comparison to the enthusiasm which prevailed everywhere when it was known that official news of the surrender of Cronje had come. For days it had seemed impossible that the Boer leader could hold out against the continuous shelling of the British forces, and yet there were no tidings, so that one's heart ached for the poor brave men fighting against impossible odds, and still more for the women and children who appeared to have formed part of the camp. It is difficult to understand why the offer of Lord Roberts to allow these non-combatants to pass through his lines earlier in the contest should have been refused, unless it was so very unlike Cronje's own behaviour at Potchefstroom. On that occasion he declined to allow the British women and children, with the exception of four, to come out at all, and I suppose he felt ashamed to accept a kindness which he himself, in like case, had persistently denied. There was something pathetic in the request made by the captured chief that he might be treated kindly. Surely he did not believe that, though we had been compelled more than once to protest indignantly against his want of honour, we should choose the present time for paying off old scores. Evidently the Boers have yet to learn that Englishmen don't generally hit a man when he's down. Was not that a nice little sentence in "Bobs'" first telegram announcing the surrender, "I hope Her Majesty's Government will consider this event satisfactory, occurring as it does on the anniversary of Majuba"? Such a delicious mixture of modesty and pride! General Buller has also scored a signal success, and has assaulted and carried the top of Pieter's Hill, taking about sixty prisoners and scattering the enemy in all directions. He is now apparently so near to Ladysmith that the welcome intelligence of its relief may arrive at any moment. There is a further list of casualties, which has already been very heavy. But no victory is bought without price, and the fact of the unconditional surrender of so brave a soldier as Cronje should do much to dishearten the rebels and bring about the close of that "terrible war," as the Princess of Wales called it the other day when she was visiting the wounded in the ship which bears her name.

Private letters from Natal by the last mail are naturally pitched in a most mournful tone, for at the time of writing General Buller had just withdrawn his troops across the Tugela. One young fellow who, with his corps, was stationed all night on the edge of Spion Kop ready to reinforce those on the top, says: "Sleep was impossible. The rain poured down all night; we had no blankets, and the sides of the mountain were so steep that it was difficult to find a spot where we could stand and keep our equilibrium. Through all the hours of darkness the stretcher bearers were going up and down, fetching their suffering burdens; the jolting for the wounded must have been terrible. Many had to be left on the mountain plateau for twelve to twenty-four hours before the ambulance could reach them. Our disappointment when the men returned from the top of Spion Kop it is impossible to describe, and as for the poor chaps themselves, they just laid down and never uttered a word, they were too heart-broken for speech. They are still of opinion that with reinforcements they could have held the position—but that is over now. Meanwhile poor Ladysmith waits on. We hear that spirits and drugs have given out, and one fellow whose sister is, he knows, very ill of enteric within the besieged town, has sent a native runner with brandy to try and get through the Boer lines. But of course he has no power of finding out whether the precious fluid ever reaches her." My correspondent continues: "It may be true that the Boers are hard up for food in some parts, but a chum of mine who is with Lord Dundonald tells me that when they captured some Boers at Acton Holmes they found the prisoners to be well-dressed young fellows, whose horses were in much better condition than their own, and inside their saddle bags were delicious roast fowls and fresh bread. Can't you imagine how quickly onr men disposed of that little lot, after having had nothing for days but biscuits and bully beef! A testimony to the efficiency of their hospital arrangements is given by a surgeon-major who managed to escape from Dundee. He says they have all the latest conveniences and up-to-date improvements, and have taken the wise precaution of painting their ambulance trains entirely white, with red crosses all over the carriages, so that even at a considerable distance they are easily recognisable as the temporary homes of the wounded. Had our own hospital trains and ambulance waggons been more distinctive there would have been far less trouble in preventing the Boers firing upon them. The distances are often so great that it is said to be difficult to note, in the whirl of the battle, unimportant distinguishing marks."

Notwithstanding the opposition which was offered in many quarters to the establishment of an Artists' War Fund, the scheme by which the brethren of the brush proposed to aid the sick and wounded has been carried through to a successful termination, and the largest sale room of Christie and Manson was crowded to overflowing on Saturday, when the first portion of the collection of pictures, water-colour drawings, etchings, and bronze and metal work was offered for sale. The Royal Family showed their interest in the undertaking not only by making purchases, but by sending contributions. The Prince of Wales bought "A Shadow of the Future," by Lady Alma-Tadema, for 100 guineas; "Bad News," by J. H. Lorrimer, R.S.A., for £50; and a bronze of the late Lord Leighton, by Thomas Brock, R.A., for 50 guineas. Of the two original etchings by the Queen, one of the mother of the German Empress fetched 180 guineas, and the other, Prince Alfred of Saxe-Coburg, 100 guineas. An etching by the Prince Consort, with an autograph specially written by Her Majesty, realised 34 guineas; two pictures by the Empress Frederick, £70 and £31 10s. respectively; and a sketch by Princess Louise, 44 guineas. The result of the first day's sale, which included canvases from many of our most celebrated artists, realised £7,359, and on Monday the total was nearly raised to £10,000, a most handsome contribution from a class who, as a rule, are none too well paid themselves.

Those who are fond of Shakespeare will have ample opportunity of gratifying their tastes during the next few weeks, for Mr. F. R. Benson, whose name is so well-known in connection with Shakesperean representations, has established himself at the Lyceum with an excellent company, and changes his programme frequently. Mr. Benson opened his season with Henry V., which met with especial favour, partly because of its strong martial element, but "A Midsummer Night's Dream," which succeeded the seldom-acted historical play, apparently met with equal success. This was the more remarkable because Mr. Tree, having produced the same play at Her Majesty's Theatre with the idea of keeping it on the bill as long as possible, was able to mount it with much more magnificent scenic display. But so admirable is the "all round" acting at the Lyceum, so poetical is the treatment accorded to the dream-like play, and so evenly is the balance kept between fantasy and buffoonery, that those who failed to witness the play at one house because they had seen it at the other have lost a good deal. "Hamlet" is to be performed alternately with "The Rivals"—the only non-Shakesperean play which the season includes—during the next fortnight, and will be given in its entirety, lasting from half-past three till about ten, with an interval for dinner. However enjoyable the performance may be, I fear that after all those hours of murder, mania, and suicide, the playgoers, as well as the play-actors, will be a little weary.

### Everybody's Opinion.

[Correspondence on all subjects is invited, but we cannot in any way be responsible for the opinions expressed by our correspondents. No communication can be entertained if the name and address of the correspondent is not given, as a guarantee of good faith but not necessarily for publication, or unless one side of the paper only is written on.]

### THE AGE LIMIT.

"E. B." writes: May I say how rejoiced I was to read Miss Mary Gardner's strong article on "The Age Limit"? "Candidates over thirty-five need not apply." What an iniquitous and wrongheaded stipulation to make! One can scarcely conceive of it as a serious fact, so obvious is it that a fine character and healthy physique will scarcely culminate in its best till some two score years and ten have run. A peevish and neurotic temperament can be seen at a glance by a committee of any discernment. But these signs do not of necessity spell years beyond the limit. No, these defects may be found finely developed well within the narrow span and are extremely likely to beset all but the most fit, who have responsibility beyond the power of their years on their shoulders. May I say that what seems to be the real secret of this "limit" never occurred to me in all my wonderings? Miss Gardner's article came quite as a revelation. She doubtlessly has given the subject much thought, and one feels through all the letter that she has gathered to herself incontestable facts; all of which but makes the situation the more preposterous. Of a surety it is horrid to grow old in body; with the dear human nature still green within us, how can we feel any other? Colours fade, locks grow scanty. Alas! it is all too visible to the man in the street! But he, good man, looks with a lenient eye; we do but go with him the way of all flesh. "God made them to match the men"—three-score years of earnest endeavour, and ten for retrospect and rest from their labours. That's what their hearts and minds and bodies are good for, if treated aright. No distinction, if you please! "They were made to match the men." How well I remember hearing our dear old (?) matron of fifty say, during a very trying sick season, that she would like only to engage women over forty as nurses and maids, because, as she said, "they are so much stronger and braver." Whence came this morbid, grisly power of the invisible? Healthy women are ashamed of this bogey; they will have none of it. Dame Nature has taken offence at it. The profession has wrenched aside the decent covering of her secret workings, and made them to become a stumbling-block unto our feet. Let the profession beware, lest an evil day befall it. Concerning the "commercial spirit," I should like to send "greeting" to the "Ex-paying Pro.," whose letter was truly beautiful, and said all that heart could desire. Oh, that she had been my pro., or I hers!

### THE MONOTONY OF PRIVATE NURSING.

"Sympathy" writes: In all ages the human conscience has been ready to excuse itself against the upholding of a higher standard of life, and people are too ready to excuse any laxity in themselves by comparison with others who, to their minds, seem more careless. This kind of comparison is fatal to conscientious thoroughness in any work. Perhaps Florence Nightingale's "Admirer" would probably find the work of an accident ward in one of our large hospitals more congenial than private nursing, for even in the "acute forms of disease" which she mentions, after the excitement of danger met, and possibly averted, by skilled treatment, there follows, of necessity, the period of convalescence, which would doubtless be "uninteresting" to one of her temperament, and the isolation imperative in all infectious cases she would probably find extremely monotonous. But if work be conscientiously undertaken and remuneration accepted, surely small duties are as incumbent upon a nurse as the more interesting ones necessary in a grave case. It would be grievous for suffering humanity if some doctors, as well as some nurses, had an indisposition to use their skill for the smaller ailments to which flesh is heir, and which, if ignored, too often lead to grave ill-health. If those who really love the work for its highest ends would join in an effort to raise it above adverse criticism, they would soon conquer the spirit of discontent which is otherwise not unlikely to become infectious.

"An Old Nurse" writes: I cannot help sympathising to some extent with "An Admirer of Florence Nightingale," because I think she is one of those energetic people who enjoy having to "fly round," and who is never happier than when she has a "good case," and feels she has her hands full. But she should remember that we cannot always have "interesting" and acute cases (a good thing, as the strain would be too great), and when we have a "monotonous" one perhaps we have something else to learn besides nursing. There was One who said: "He that is faithful in that which is least, is faithful also in much." So, unless we are content to do our best in the dull cases, we may fail in the others.

### The Conversazione at the London Hospital.

One of the most popular functions of the year at "The London" is the entertainment given at the Medical College by the Musical Society, to which the entire nursing staff are invited, and which is always a great occasion for a "gathering of the clans," so to speak, on the part of old Londoners. The college lends itself well to such a purpose, and provides ample space even for the many who invariably seize this opportunity of meeting old friends and spending a pleasant evening. The guests, who fairly thronged the college on Thursday last week, found a varied programme provided for their amusement, and medical and nursing staffs, past and present, well represented. Amongst others should be mentioned Dr. and Mrs. Sansom, Dr. and Mrs. Herman, Mr. and Mrs. Openshaw, Dr. Gilbart Smith, Dr. and Mrs. Percy Kidd, Mr. J. Hutchinson, jun., and Mrs. Hutchinson, and Dr. and Mrs. Fred Smith. Mr. Sydney Holland was there, and Mr. Douro Hall, Mrs. Spender, Mr. G. Q. Roberts, Miss Bland, and many others, while Miss Lückes must have found it hard to greet all who wanted a word with "Matron." The band of the Royal Artillery played "sweet, stirring, and patriotic melodies" in the Library throughout the evening; an excellent concert attracted large audiences in the Athenæum; there was a most interesting exhibition of Dr. Gilbart Smith's photographs; as well as demonstrations of "wireless telegraphy," and X-Rays, with palmistry in the seclusion of the Materiæ Medica Museum. For those who do not have enough of them at other times a choice assortment of bacilli was on view in the Biological Laboratory; while "The Story of a Match," illustrated with chemical experiments, was told by Mr. F. Page, B.Sc.Lond., in the Chemical Theatre. A cinematograph show was a feature of the evening; and last, but by no means least, thorough justice was certainly done to the admirable refreshments provided. Altogether the conversazione of 1900 may be written down as a distinct success, on which Mr. H. Balean and Mr. E. M. Perkins, hon. secretaries of the Musical Society, are cordially to be congratulated. It must be added to the list of pleasant gatherings at the London, so thoroughly enjoyed by the many who are bound to the great hospital by ties of long association and warm interest.

### For Reading to the Sick.

The sacrifice of God is a troubled spirit, a broken and a contrite heart, O God, shalt Thou not despise.—*Ps.* li. 17.

In repentance too is man purified. It is the grand Christian act. . . . Of all acts is not for man repentance the most divine?—*Carlyle.*

In trouble for my sin, I cried to God,
To the Great God who dwelleth in the deeps;
The deeps return not any voice or sign,
But with my soul I know Thee, O Great God;
The soul Thou givest knowest Thee, great God,
And with my soul I sorrow for my sin.
Full sure I am there is no joy in sin;
Sin is established subtlely in the heart,
As a disease; like a magician foul
Ruleth the better thoughts against their will,
Only the rays of God can cure the heart,
Purge it of evil; there's no other way
Except to turn with the whole heart to God.
—*Allingham.*

**Reading.**

There is a practical thought for the especial season of Lent. If Christ voluntarily became a man of sorrows, for His whole life on earth, on account of our sin, shall we refuse, for a small part of a year, to make ourselves sorrowful for our own sinfulness? and that, in order to draw nearer to Him? Can we shrink from denying ourselves for His sake, Who so utterly denied Himself for us? and think it unreasonable to withdraw for a few weeks from the world, when He gave up, not only heaven, but even such joys as He might have found here, for our advantage?

Let His awful sorrowfulness, His unutterable grief, move us to give ourselves wholly to Him. Watching Him, through this season, pass along His way of sorrows to His heart-broken death, shall we not say, with His loving disciple of old, "Let us also go, that we may die with Him"—ay, die to sin, to the world, to self, to all that is not of Him?—*St. H. M. Bourne.*

To some of us the season of Lent has come finding us suffering, sorrowful, bereaved. Bowed down under the dumb weight of an anguish "against which we dare not cry," laid aside for the present from all active participation in the service of our Lord, we would yet unite ourselves with those who at this season join themselves more closely to Him. For, unable as we are to take part in visible service, there is open to all helpless sufferers a way by which they can join those who "wait upon God." From our bed of sickness, from the depths of a lonely heart, whether it be in our own chamber or a crowded hospital ward, we can send up the silent intercession for those who are suffering, it may be, more grievously than ourselves. We can ask the Father of Mercies that He in His great pity will have compassion on the sick, the sorrowful, the desolate; on those who are out of the way; on the tempted, the tried, the fallen; on our soldiers and sailors, and all those who on the field of battle lie "in darkness, and the shadow of death." Then no longer shall we feel helpless and alone, when by our prayers we unite our sufferings to those of the afflicted and distressed. And from whatever cause we have been "called apart," then in patience we will await His good pleasure, realising that we can serve Him, even if it be for the present His will that we should only "stand and wait."—*F. B.*

### To Nurses.

We invite contributions from any of our readers, and shall be glad to pay for "Notes on News from the Nursing World," or for articles describing nursing experiences, or dealing with any nursing question from an original point of view. The minimum payment for contributions is 5s., but we welcome interesting contributions of a column, or a page, in length. It may be added that notices of entertainments, presentations, and deaths are not paid for, but, of course, we are always glad to receive them. All rejected manuscripts are returned in due course, and all payments for manuscripts used are made as early as possible at the beginning of each quarter.

### Notes and Queries.

The Editor is always willing to answer in this column, without any fee, all reasonable questions, as soon as possible.

But the following rules must be carefully observed:—

1. Every communication must be accompanied by the name and address of the writer.
2. The question must always bear upon nursing, directly or indirectly.

If an answer is required by letter a fee of half-a-crown must be enclosed with the note containing the inquiry.

*L.O.S.*

(216) I am anxious to pass L.O.S., but being a district nurse have only a short time to do it in. Where had I better apply, and what is the expense to be considered? Is certificate in monthly nursing considered enough for a district nurse, or must it be in midwifery? Where could I get particulars of the Holt-Ockley cottage nursing system?—*Nurse Marguerite.*

It is better for a district nurse to take the L.O.S., although one for monthly nursing is often considered a sufficient qualification. Write to the Secretary, the London Obstetrical Society, 20, Hanover Square, W., as you might be locally prepared for their examination, which would much lessen the cost. The address of the Holt-Ockley Association is 12, Buckingham Palace Road, S.W.

*Completion of Certificate.*

(217) Miss E. E. was trained for 18 months in the Seamen's Hospital, Greenwich, and then, through ill-health (gastric ulcer), she was obliged to leave. This was in 1894. She is now in good health, and is desirous of entering a London hospital for three years' training. Will the Editor kindly inform her if the West London Hospital, the Metropolitan Hospital, and the Great Northern Central Hospital are good training schools, and which, if any, is the best?

Any of the above-mentioned hospitals grant three years' certificates. Post-graduate classes are arranged for medical men at the West London Hospital, and as the hospital has no medical school the nurses get a large proportion of interesting work.

*Three Months' Work.*

(218) Is there any hospital (not fever) where I could take three months' further training without paying any fee? I am a lecturer on health and sick nursing of five years' standing.—*S. A. B.*

The best means of hearing of training in return for services is by advertisement.

*The Nurses' Co-operation.*

(219) Seeing a small paragraph on the success of the Nurses' Co-operation in the "Mirror," I would like very much to know what co-operation is meant, and would be so much obliged if you would give me the address. —*J. Q.*

The Nurses' Co-operation, 8, New Cavendish Street, W., is a society for the mutual benefit of nurses. It is managed by representative committees, and its members number about five hundred.

*Address.*

(220) "A. M." would be much obliged for the address of Miss Broadwood, Cottage Nurse Association.

Miss Broadwood, Holt-Ockley Nursing Association, 12, Buckingham Palace Road, S.W.

*Jaconet, &c.*

(221) Would you please let me know what is "jaconet," "red lotion," and any other name for "chlorinate of soda," mentioned in the article on "Bedsores"? I have not seen them used in my hospital.—*A. Z.*

Jaconet is a very thin waterproof material, waterproofed on one side only, which is in constant use in most hospitals for various purposes. Red lotion is an old-fashioned lotion, which used to be very much used in days before antiseptics. It is a solution of sulphate of zinc coloured rather deeply by the addition of tincture of lavender. It has what used to be called "astringent and stimulating" properties, and it was the common resort when granulations were "flabby." The Guy's formula is as follows: "Take of zinc sulphate, 2 grains; compound tincture of lavender, 10 minims; water, 1 fluid ounce; dissolve." Doubtless it is a slight antiseptic, to which fact some of its unquestioned virtues are due; which is true also of no inconsiderable number of the old balsams and aromatic wines in which our grandmothers put such faith. It is to be presumed that "Sussex" in her answer on page 251, when she spoke of "chlorinate of soda," meant "solution of chlorinated soda." This is a Pharmacopœial preparation which the dispenser will provide on the surgeon's prescription. While on the subject, let us impress upon all nurses the advantage of accuracy of language. Talking among themselves nurses, like most other people, get into slang ways; but it is a bad habit. What can be more ridiculous than to talk of fomenting a sore with "weak carbolic, or weak perchloride of mercury or chlorinated soda." What is "carbolic"? Does she mean carbolic acid, or carbolic oil, or carbolic soap? Of course she means carbolic lotion, but why does she not say so? Then, how can perchloride of mercury be weak? and how would she set to work to use a solid as a fomentation? Again of course she means a lotion; but she should say so. Laxity of language leads to laxity of thought, and also to many dangers, for nurses trained at different schools may attach very different meanings to ill-defined expressions.

*Standard Books of Reference.*

"The Nursing Profession: How and Where to Train." 2s. net.
"The Nurses' Dictionary of Medical Terms." 2s. 6d. net.
"Burdett's Series of Nursing Text-Books." 1s. each.
"A Handbook for Nurses." (Illustrated.) 5s.
"Nursing: Its Theory and Practice." New Edition. 3s. 6d.
"Helps in Sickness and to Health." Fifteenth Thousand. 5s.

All these are published by The Scientific Press, Ltd., and may be obtained through any bookseller or direct from the publishers, 28 & 29, Southampton Street, London, W.C.

### Travel Notes.

By Our Travelling Correspondent.

### XLV.—DEVONSHIRE IN WINTER.

I much prefer it in summer, but if we cannot go abroad in the winter, beautiful Devon offers us a chance of being much in the open air, and of as much sun as can reasonably be expected to shine upon us in our favoured but misty Isle.

### Dartmouth.

Torquay, Teignmouth, Dawlish, Exmouth, &c., are so well known, at least by repute, that I shall speak of less well-known places first. First in my affections stands Dartmouth. It is beautifully situated in the heart of exquisite scenery, excursions all round are easily made, and—crowning comfort—it is comparatively cheap, not as yet very fashionable, and a diligent search will be rewarded by finding reasonable lodgings. The old town itself is still extremely picturesque, though a large number of its sixteenth century buildings have been removed. Many, however, still remain, notably those in the Butter Walk. St. Saviour's Church will repay examination, the art screen, the splendid *miserères*, and the ironwork on the great door, with a quaint representation of leopards apparently hiding in the branches of a tree, are all well worth study. If sea air is objected to, Totnes is another charming place in which to stay; it is 10 miles from Dartmouth, and the steamer makes the excursion daily. The journey up the Dart is wonderfully lovely; about half-way is Dittishren, an ideal Devonshire village. The guide books dismiss its charms very curtly, but it is a place to dream of after a visit. Totnes itself is a singular old town, somewhat resembling Chester on a very minute scale, and reminding travellers of Italy. The best specimens of the arcaded houses are in the main street near the Guildhall; here, too, is the church and the north gate. Totnes makes a good place for visiting Dartmoor, though winter is hardly the time for that. Two miles from Totnes is Berry Pomeroy. The castle, erected in the days of William the Conqueror, is upon the edge of an abrupt cliff and must once have been almost impregnable. It has fallen into decay since the time of the Revolution of 1688. It is a great place for picnics and always makes a delightful place to ramble in. Brixham, Torquay, Chudleigh, and Newton Abbot may all be visited from Dartmouth, though they are rather long excursions.

### Exmouth.

This is a town that has lately been coming into considerable repute. It cannot compare in point of scenery with Dartmouth, Totnes, or Torquay, and for the town itself it is prosaic to the last degree; but it has great advantages. It is, as its name signifies, situated at the mouth of the Exe, and the sunsets are very fine. It really belongs to the parish of Littleham, where there is a pretty church. The climate is rather dryer than that of some Devonshire places, and seems well suited to elderly people whose bronchial apparatus may not be all that could be desired. Probably on account of this it has gradually become a refuge for retired officers, dignitaries of the church, &c., who have perforce given up an active part in life. One great advantage enjoyed by Exmouth is its nearness to Exeter, that most interesting and dignified of cathedral cities. Trains run frequently, so that it is easy to spend even a winter afternoon shopping and attending service in the Cathedral. Hotel accommodation is somewhat high-priced, and so are lodgings on The Beacon, which is the aristocratic corner of Exmouth, facing the sea, and enjoying a splendid view and invigorating air; but more in the town apartments are not so dear, and if visitors are not confined to the house, these localities would suit quite as well.

### Budleigh Salterton.

This very charming little watering place lies about five miles from Exmouth, and is reached by coach, which leaves early in the morning and returns in the late afternoon. Personally, I prefer it as a place of residence to Exmouth, it is not so fashionable, and though now fast becoming popular it has hitherto retained its character of village simplicity. Lodgings for a protracted stay may be had reasonably. One great advantage of Budleigh Salterton is the long stretch of nearly level walking that may be had right through the little town to the country beyond. This is a very great thing for those who cannot stand hill climbing.

### Torquay.

One cannot speak of Devonshire and entirely omit mention of Torquay, though it almost seems a superfluous undertaking, when almost everyone knows it. It is one of the prettiest modern towns in England, and its picturesqueness is greatly enhanced by the forced building of its houses and streets at every conceivable elevation and angle. This always adds to the beauty of a place, but somewhat detracts from its comfort for invalids. However, cab hire is cheap and the horses strong and to the manner born, so that, having ventured down from one's eyrie, it is easy to attain the heights again behind one of these strong little horses, who make very light of their daily climbs. It is certainly a delightful place in which to spend a winter, the walks all round including Babbicome, Ilsham, &c. The coach drive from Bovey Tracy over Dartmoor, the steamer trips to Paignton, &c., all make pleasant varieties in one's daily life. Newton Abbot, with the exquisite walk through Bradley Woods, is easily undertaken in a day, besides many other excursions too numerous to mention.

### North Devon.

I have said nothing of North Devon for it is not quite so suitable for the generality of winter visitors. All the same, for those who like a more bracing climate than that of the rather humid and relaxing south they would do well to try such places as Lynton, Lynmouth, Minehead, and Ilfracombe. The latter, however, has become of late crowded and fashionable, and so only fit for those who like to dress several times a day and enter into mild society. Since the railway reached its primitive seclusion Ilfracombe has entirely changed its character; it is still a beautiful place, and for gay souls with plenty of money I do not know a more suitable resort. In the winter Lynmouth and Lynton are, to a certain extent, cut off from the outside world because most of the coaches cease to run, and then many people find it dull, unless they made up a party among themselves. There are many places a little inland that would be cheap and pleasant in winter and early spring. The neighbourhood of Barnstaple abounds in such spots, and in many way they are decidedly preferable to the seaside in chilly weather.

### TRAVEL NOTES AND QUERIES.

French Riviera (Pigeon).—I think you need have no fear. I am sure the feeling is greatly exaggerated; I have friends now living both at Nice and Cannes, and they say they have experienced nothing of this hostile feeling. I think it would be much more dignified if we took no notice of such publications as have lately appeared in France, and treated them as being beneath contempt. It is not Frenchmen generally who feel towards us like this, only an insignificant minority.

Verona (Artist).—Under the circumstances I should not choose Verona till late in the year; it is terribly cold, and you would be ruined in endeavouring to warm your Arctic rooms; it is a lovely city, but not really comfortable till April and May. Venice is still colder, because the sun cannot penetrate far into its narrow canals. Why not try Florence. Though cold, it is so sunny that one does not feel it in the same way. I am told Siena is pleasant in winter, but it is also too cold for my taste. I was there once in November, and found it very trying; the same reason holds good, the sun cannot penetrate the high narrow streets.

## Figures and Tables

**Figure f1:**